# Prevalence of infectious bursal disease in chicken populations in China during 1983–2024: a meta-analysis

**DOI:** 10.3389/fvets.2026.1534532

**Published:** 2026-02-23

**Authors:** Mingfeng Chu, Junxue Qiu, Huiying Zhang, Shuiyun Chen, Yuchen Liang, Mengke Si, Yiwei Wang, Wei Cheng, Baolei Yang, Xiaoyu Chong, Xuelong Chen, Yanping Qi

**Affiliations:** 1 Anhui Province Key Laboratory of Animal Nutritional Regulation and Health, Anhui Science and Technology University, Fengyang, China; 2 Anhui Engineering Technology Research Center of Pork Quality Control and Enhance, Anhui Science and Technology University, Fengyang, China

**Keywords:** infectious bursal disease, China, chicken populations, epidemic, meta-analysis

## Abstract

**Importance:**

Infectious bursal disease (IBD) is an acute, highly contagious and immunosuppressive disease caused by the Infectious bursal disease virus (IBDV), which has brought significant economic losses to China's chicken industry. This meta-analysis aimed to evaluate the prevalence of IBD among in chicken populations across mainland China.

**Methods:**

A total of 32 studies on infectious bursal disease virus in China were identified through a comprehensive search of six major databases: PubMed, Google Scholar, Cochrane Library, VIP, China National Knowledge Infrastructure (CNKI), and Wanfang. These publications, spanning 1983–2024, were used to estimate the prevalence of infectious bursal disease in chicken populations. Based on predefined exclusion criteria studies were excluded if they were duplicates, involved non-chicken hosts, or lacked sufficient data to calculate prevalence rates in chickens in mainland China. Relevant data were then extracted from the remaining studies that provided estimates of IBDV infection prevalence in chicken populations across the region.

**Results:**

A total of 32 studies encompassing data from 210,983 chickens met the study criteria for analysis. The pooled prevalence of infectious bursal disease infection was estimated at 37% (95% CI: 29–46). The prevalence in Northwest China was significantly higher, reaching 88% (95% CI: 85–91), considerably exceeding rates observed in other regions. The prevalence of IBDV infection was associated with the region, type, growth stage, scale, published time, season, and detection method.

**Conclusion:**

The prevalence of IBD displays clear seasonal and regional patterns. Therefore, ongoing surveillance of IBDV and the development of targeted control measures tailored to specific conditions are essential. Further, effective and well-coordinated interventions are crucial to limiting the spread of IBDV among chicken populations, particularly during periods of low temperature and favorable humidity, when transmission risk is high.

**Systematic review registration:**

https://www.crd.york.ac.uk/prospero/display_record.php?ID=CRD420261296958, identifier: CRD420261296958.

## Highlights

- The data of 32 articles met the selection criteria, and the prevalence of IBDV was 37% in China.
- Regions of chicken populations was a significantly risk factor associated with the prevalence rate of IBDV.
- This study may provide valuable advice for policymakers, veterinarians, and stakeholders in the chicken industry, offering an evidence base to formulate appropriate guidance for the prevention and control of IBDV.

## 1 Introduction

Infectious bursal disease (IBD) is caused by the infectious bursal disease virus (IBDV), a double-stranded RNA virus classified within a genus of avian viruses in the dsRNA virus family. The disease is clinically characterized by signs such as lethargy, reduced appetite, white watery diarrhea, and pathological changes in the bursa of Fabricius, including swelling, hemorrhage, and eventual atrophy (1, 2). IBDV spreads through direct and indirect contact, with chicks between 3 and 6 weeks of age particularly vulnerable (3). The disease usually presents with co-infection or secondary infection, with a mortality rate typically ranging from 30% to 60% and reaching 80% during severe outbreaks (4). Infectious bursal disease was first detected and documented in North America in 1957. Since then, it has rapidly spread worldwide, reaching Europe, Asia, Africa, and South America (5).

In China, IBD was first identified in 1979 in the provinces of Beijing and Guangdong, after which it quickly spread to all chicken-producing regions across the country (6). Since its initial detection, IBD has been reported in every province, with evident temporal and geographic fluctuations in its incidence (7). For instance, in 1981, the incidence rate in Shanxi province reached 75% with an associated mortality rate of 40% (8). A seroprevalence study conducted on 29,680 chickens in Ningxia, which employed the enzyme-linked immunosorbent assay (ELISA), revealed an incidence rate of 1.56% and a mortality rate of 32.61% (9). In 2020, 12 IBDV strains were recently isolated from chickens in Hubei province, resulting in substantial economic losses to the Chinese chicken industry (10). Although detection rates vary by region, the prevalence of IBDV- positive samples in most areas has ranged between 17.33% and 41.46% (11). As a result, numerous studies have investigated the prevalence of IBD in China, offering valuable insights into the epidemiological profile of IBDV across the country.

Given the relatively fragmented nature of epidemiological studies on chicken IBD in China, this study employed a meta-analysis approach to systematically evaluate the relevant literature on the disease's epidemiology from 1983 to 2024. Additionally, the study examined potential risk factors associated with IBD infection in chicken populations by statistically analyzing data related to geographic region, type, growth stage, farming scale, sampling year, season (Spring: March–May, Summer: June–August, Autumn: September–November, Winter: December–February), and detection method. The goal was to clarify trends in IBD prevalence and inform the development of targeted prevention strategies, while also offering a foundation for future research on IBD in chickens.

## 2 Methods

### 2.1 Search strategy

Following the PRISMA guidelines (12) we identified epidemiological studies on IBD conducted in China and published in either Chinese or English between January 1, 1983, and January 1, 2024. Relevant literature was retrieved through searches of English-language databases (PubMed, Google Scholar, Cochrane Library) and Chinese-language databases (VIP, CNKI, Wanfang Data) used search terms included combinations of the following keywords: “infectious bursal disease” or “infectious bursal disease virus,” “epidemiology,” “incidence” or “prevalence,” “chicks,” or “adult chickens,” and “China” or “mainland China.”

### 2.2 Data management

Two investigators independently evaluated the extracted data to determine whether they met the inclusion criteria, and studies that met the requirements were ultimately included. In cases of discrepancies during the screening process, expert consultation, and analysis were sought to determine study inclusion. This research involved a statistically controlled primary survey where data were extracted directly from the articles without further verification from the authors. The study also did not include unpublished data.

### 2.3 Study selection criteria

A preliminary screening was conducted on studies covering IBDV in Chinese chicken populations between January 1983 and January 2024, with the inclusion criteria as follows: (1) epidemiologic studies focusing on chicken populations in various provinces of China, (2) studies with >10 samples (to ensure reliability), (3) including the statistical analysis of data related to region, type, growth phase, scale, sampling year, season (Spring: March–May, Summer: June–August, Autumn: September–November, Winter: December–February), detection method, prevalence-based data.

### 2.4 Exclusion criteria

Studies were excluded if they were (1) reviews, (2) studies of animals outside of China, (3) incomplete or contained duplicate data, (4) focused on other pathogens or hosts.

### 2.5 Data extraction

Data were extracted for inclusion in the papers using a standardized data collection form containing the following information: first author, region, type, growth phase, scale, sampling year, season (Spring: March–May, Summer: June–August, Autumn: September–November, Winter: December–February), detection method, and total number of tested chickens and the number of IBD positive chickens.

### 2.6 Analysis of study quality

Study quality was assessed using the adapted Newcastle-Ottawa Scale (NOS) (13). The scoring for each subgroup was determined based on criteria that including the study's stated purposes, detection method, sampling time, sample collection details, and risk factor analysis. A maximum of 5 points could be awarded for each study. A total score of 1 was considered low quality, 2–3 was considered moderate quality, and 4–5 was assigned as high quality.

### 2.7 Statistical analysis

A meta-analysis was performed using Stata 12 software (Stata Corp. College Station, Texas) to calculate the overall prevalence of IBD infections in chicken populations and to assess heterogeneity. The results (including 95% confidence intervals) are presented as a forest plot. Heterogeneity was evaluated using the I^2^ and Cochrane's Q (denoted as c2 and *p-value*) statistics, and heterogeneity was analyzed using a random effects model, with statistically significant heterogeneity in the results of the trials when *I*^2^ > 50% and *p* < 0.5 as detailed in Table 2. In the present study, different factors affecting heterogeneity were analyzed separately and a multivariate model was developed that examined geographic region, type, growth stage, farming scale, sampling year, season, detection method, and assessed the mean chicken population size. Funnel plots were employed to assess potential publication bias among the included studies. A *p-value* of less than 0.05 in the Egger test indicated significant publication bias, while a *p-value* of 0.05 or higher suggested non-significant (6).

## 3 Results

### 3.1 Search results and eligible studies

**Figure 1:**
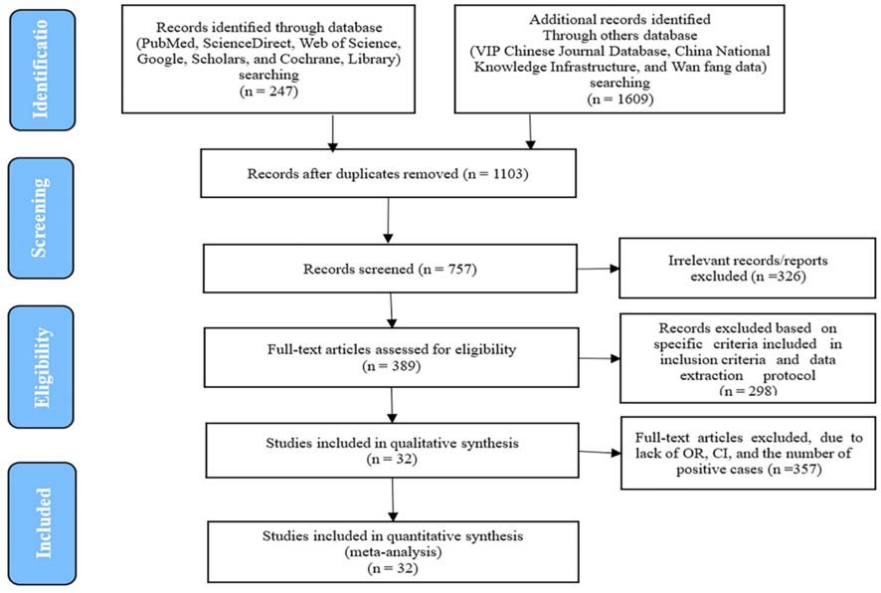
PRISMA flow diagram of the literature search, screening, assessing for eligibility, and selecting articles for the meta-analysis.

**Figure 2:**
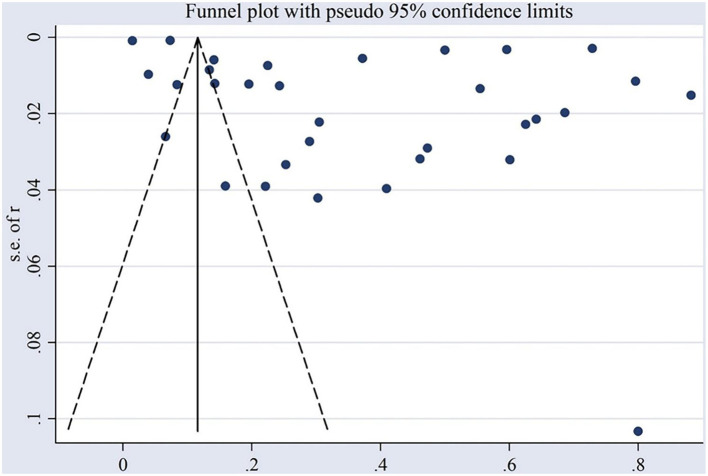
Funnel plot with pseudo 95% confidence interval.

This meta-analysis evaluated the prevalence of IBDV in chicken populations across China. An initial search of relevant databases yielded 1,429 articles. After removing duplicates and conducting a preliminary screening, 32 studies met the inclusion criteria (Figure 1). These studies, published between 1983 and 2024, covered data from 19 provinces and included a total of 210,983 chickens, among which 58,905 tested positive for IBDV (Figure 2). A cross-sectional analysis was performed across all included studies, to calculate prevalence rates over the study period. Based on the established quality assessment criteria, 11 studies were rated as high quality (4–5 points), 17 as moderate quality (2–3 points), and 4 as low quality (1 point) (Table 1). The funnel plot revealed asymmetry in the overall IBDV prevalence data, indicating a potential publication bias in the included literature (Figure 2).

**Table 1:** Summary of studies on IBDV infection in chickens in mainland China.

**References**	**Province**	**Region**	**No. examined**	**No. positive**	**Prevalence**	**Detection method**	**Study design**	**Quality score**
Bai (45)	Jilin	Northeast China	15	12	80%	AGP	Cross-sectional	4
Dai (46)	Guangxi	South China	113	25	22%	RT PCR	Cross-sectional	2
Feng (47)	Anhui	East China	451	282	63%	ELISA	Cross-sectional	1
Hua et al. (48)	Jilin	Northeast China	430	131	30%	AGP	Cross-sectional	4
Huang (49)	Fujian	South China	500	42	8%	ELISA	Cross-sectional	3
Ji et al. (50)	Jiangsu	East China	405	16	4%	ELISA	Cross-sectional	2
Jiao (51)	Guangxi	South China	88	14	16%	AGP	Cross-sectional	3
Lan et al. (52)	Guangxi	South China	170	43	25%	ELISA	Cross-sectional	3
Li et al. (53)	Henan	Central China	17,287	250	1%	AGP	Cross-sectional	4
Liao et al. (54)	Guangdong	South China	119	36	30%	ELISA	Cross-sectional	5
Lin et al. (55)	Fujian	South China	7,575	2,817	37%	ELISA	Cross-sectional	5
Liu et al. (56)	Anhui	East China	233	140	60%	ELISA	Cross-sectional	1
Qiao et al. (57)	Shanxi	North China	1,360	754	55%	ELISA	Cross-sectional	2
Qin et al. (58)	Guangxi	South China	245	113	46%	ELISA	Cross-sectional	2
Qiu et al. (59)	Hebei	North China	3,477	491	14%	AGP	Cross-sectional	2
Wang et al. (60)	Anhui	East China	551	378	69%	ELISA	Cross-sectional	2
Wang (61)	Shandong	East China	829	118	14%	ELISA	Cross-sectional	4
Wu et al. (62)	Neimenggu	North China	3,200	719	22%	AGP	Cross-sectional	3
Wu et al. (63)	Henan	Central China	97,326	7,134	7%	AGP	Cross-sectional	3
Xiao et al. (64)	Jiangxi	East China	1,225	975	80%	AGP	Cross-sectional	4
Yang et al. (65)	Hubei	Central China	21,800	10,900	50%	AGP	Cross-sectional	3
Yang et al. (66)	Anhui	East China	154	63	24%	RT PCR	Cross-sectional	1
Yuan et al. (67)	Zhejiang	East China	24,027	17,512	73%	ELISA	Cross-sectional	2
Cui et al. (68)	Heilongjiang	Northeast China	24,000	14,300	60%	ELISA	Cross-sectional	5
Zhang et al. (69)	Henan	Central China	1,605	215	13%	AGP	Cross-sectional	2
Zhang and Han (70)	Neimenggu	North China	296	140	47%	AGP	Cross-sectional	5
Zhang (71)	Henan	Central China	91	6	7%	ELISA	Cross-sectional	2
Zhen et al. (72)	Heilongjiang	Northeast China	1,050	205	20%	AGP	Cross-sectional	4
Zhong et al. (73)	Guizhou	Southwest China	1,135	276	24%	AGP	Cross-sectional	2
Zhou et al. (74)	Hainan	Central China	500	321	64%	AGP	Cross-sectional	3
Zhou et al. (75)	Shanghai	East China	276	80	29%	ELISA	Cross-sectional	1
Zhou et al. (17)	Ningxia	Northwest China	450	397	88%	ELISA	Cross-sectional	5

### 3.2 IBDV prevalence in different regions of China

**Figure 3:**
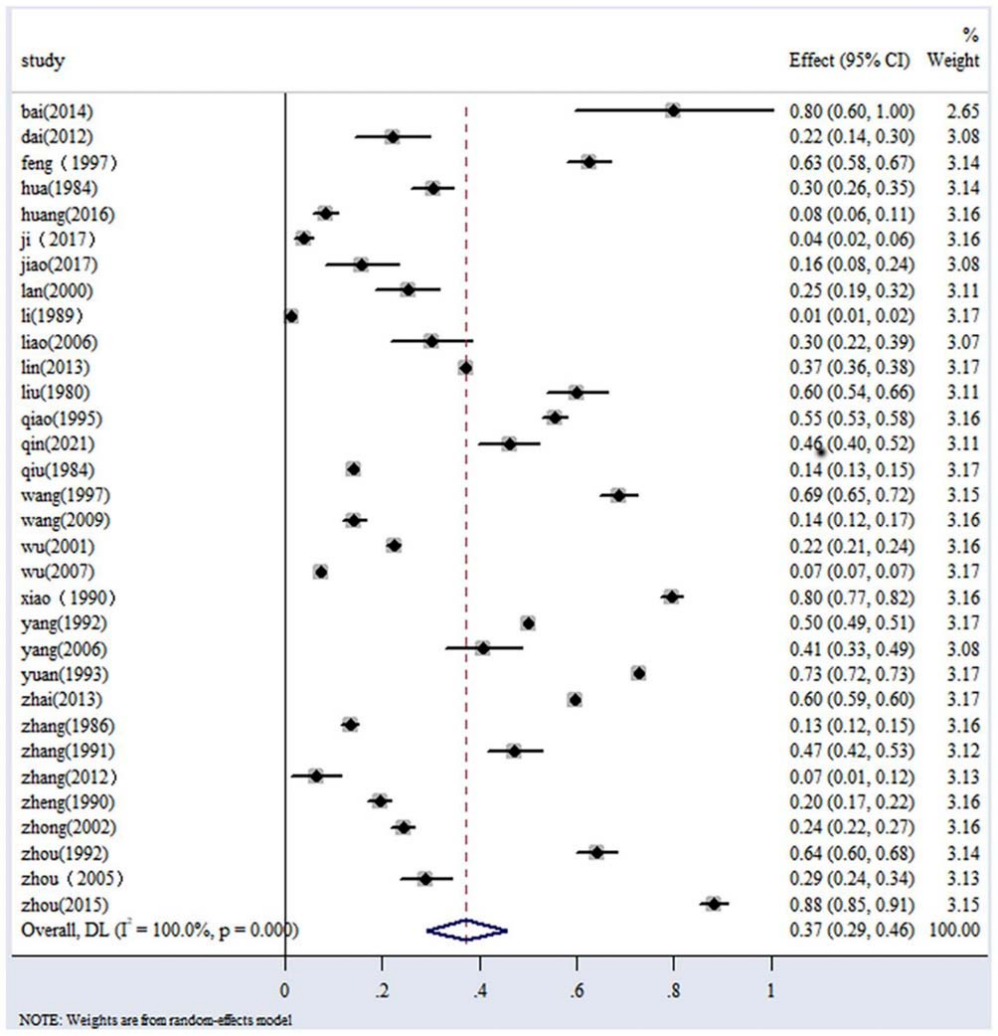
Random effect meta-analysis of avian IBDV infection in Chinese mainland.

**Figure 4:**
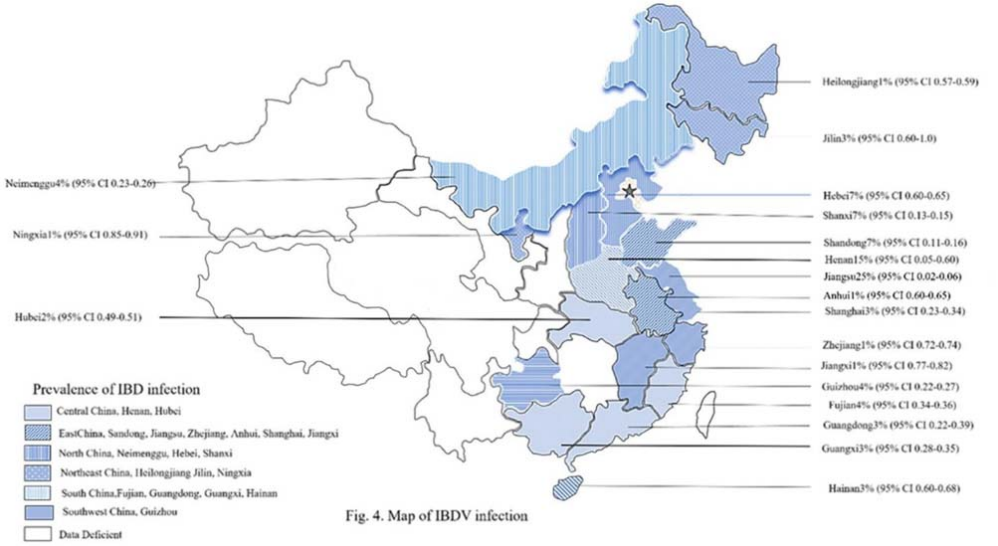
Distribution of IBDV infection rate by provinces in mainland China.

The reported prevalence rates of IBDV among chicken populations in the included studies varied widely, ranging from 1% to 88% (Table 1, Figure 3). Across all regions of China, the overall estimated prevalence of IBDV was 37% (95% CI: 29–46), based on data from 210,983 samples (Table 2, Figure 4).

**Table 2:** Overall prevalence of IBDV infection in chickens in mainland China.

**Subgroup category**	**Subgroup item**	**No**.	**No**.	**No**.	**Crude prevalence**	**% (95% CI)**	**Heterogeneity**		
		**Studies**	**Tested**	**Positive**			*χ^2^*	***p-value***	***I**^2^* **(%)**
Region	Northeast China	4	25,495	14,648	57%	46% (20–73)	1,151.12	0.000	99.27%
	North China	4	8,333	2,104	25%	35% (18–51)	870.0	0.000	99.7%
	Northwest China	1	450	397	88%	88% (85–91)	0	0.000	0.0%
	East China	9	28,151	19,564	69%	48% (25–71)	6,862.09	0.000	99.9%
	South China	8	9,310	3,411	36%	31% (18–44)	709.62	0.000	99.0%
	Central China	5	138,109	18,505	13%	16% (6–26)	19,722.55	0.000	100.0%
	Southwest China	1	1,135	276	24%	24% (22–27)	0	0.000	0.0%
Sampling year	>10 years	10	33,482	17,742	53%	36% (20–53)	5,867.12	0.000	99.8%
	<10 years	22	177,501	41,163	23%	38% (29–47)	79,915.51	0.000	100.0%
Type	Layer chickens	8	70,404	33,523	48%	39% (12–66)	84,177.24	0.000	100.0%
	Broiler chickens	15	13,570	3,988	29%	31% (24–39)	11,508.53	0.000	98.9%
Growth phase	Other	9	12,7009	21,394	17%	41% (24–58)	21,640.06	0.000	100.0%
	Newly hatched chickens	9	31,955	5,484	17%	33% (15–50)	10,352.67	0.000	99.9%
	Young chickens	5	99,045	7,446	7%	11% (5–18)	194.70	0.000	97.9%
	Adult chickens	4	2,566	1,007	39%	31% (6–51)	659.69	0.000	99.5%
	Other	14	77,417	44,968	58%	52% (40–63)	12,046.21	0.000	99.9%
Detection method	AGP	15	149,667	21,923	15%	34% (27–41)	26,101.15	0.000	99.9%
	ELISA	15	61,049	36,894	60%	41% (29–52)	11,227.36	0.000	99.9%
	RT PCR	2	267	88	32%	32% (13–50)	11.40	0.001	91.2%
Scale	Intensive chicken farm	7	128,741	21,694	17%	45% (24–66)	20,872.61	0.000	100.0%
	Free-range chickens	10	30,484	16,896	55%	42% (25–58)	5,000.50	0.000	99.8%
	Other	17	51,758	20,315	39%	35% (15–54)	59,038.48	0.000	100.0%
Season	Spring	4	1,656	650	39%	39% (2–80)	1,684.09	0.000	99.8%
	Summer	2	3,496	859	25%	35% (10–59)	68.76	0.000	98.5%
	Autumn	2	45,800	25,200	55%	55% (45–64)	427.13	0.000	99.8%
	Winter	3	24,877	3,079	12%	38% (8–67)	4,091.39	0.000	100.0%
	Unknown	21	135,154	29,117	22%	36% (20–51)	54,979.56	0.000	100.0%
Total		32	210,983	58,905	28%	37% (29–46)		0.000	100.0%

The highest IBDV prevalence was reported in northwestern China at 88% (95% CI: 85–91), followed by east China at 48% (95% CI: 25–71), northeast China at 46% (95% CI: 20–73), north China at 35% (95% CI: 18–51), south China at 31% (95% CI: 18–44), and southwest China 24% (95% CI: 22–27). The lowest IBDV prevalence was observed in central China at 16% (95% CI: 6–26) (Table 2).

### 3.3 Factors associated with reported IBDV prevalence

We examined various risk factors linked to the prevalence of IBDV infection in chickens. The prevalence of IBDV before and during 2010 was estimated at 38% (95% CI: 29–37), while the rate declined slightly to 36% (95% CI: 20–53) after 2010, indicating a higher infection burden before 2010 (Table 2). When stratified by diagnostic method, ELISA yielded a prevalence of 41% (95% CI: 29–52), compared to 34% (95% CI: 27–41) with AGP, and 32% (95% CI: 13–50) with RT-PCR.

Regarding seasonal patterns, IBDV prevalence peaked in autumn (September–November) at 55% (95% CI: 45–64), followed by spring (March–May) at 39% (95% CI: 2–80), winter (December–February) at 38% (95% CI: 8–67), and summer (June–August) at 35% (95% CI: 10–59) (Table 2). In chicken populations of different ages, there are certain differences in the prevalence rate of IBDV. The prevalence rate in newly hatched chickens is the highest, reaching 33% (95% CI: 15–50), followed by that in adult chickens, which is 31% (95% CI: 6–56), and the prevalence rate in young chickens is relatively low, at 11% (95% CI: 5–18). From the perspective of chicken populations types, the prevalence rate of infectious bursal disease virus in layer chickens is 39% (95% CI: 12–66), while that in broiler chickens is 31% (95% CI: 24–39), with layers having a slightly higher prevalence rate than broilers. In terms of breeding modes, the prevalence rate of IBDV in intensive chicken farm is 45% (95% CI: 24–66), and the prevalence rate in free-range chickens is higher, reaching 42% (95% CI: 25–58).

### 3.4 IBDV and biosecurity measures

The prevalence of IBDV varies significantly among chicken populations with different biosecurity levels. Chicken populations that implement comprehensive biosecurity protocols (including regular disinfection, visitor restrictions, and all-in -out production systems) have relatively low IBDV prevalence rates, while chicken populations with inadequate biosecurity measures have significantly higher IBDV prevalence rates.

## 4 Discussion

As one of the most impactful epidemics in the chicken sector, IBDV is designated as a Category B disease by the International Veterinary Bureau (IVB) under the 1999 edition of the International Code of Animal Health (ICOA) (14). In 2024, China recorded a total chicken slaughter volume of 17.34 billion birds, marking an increase of 51 million chickens (3.1%) compared to the previous year (https://www.stats.gov.cn/xxgk/jd/sjjd2020/202501/t20250117_1958344.html), underscoring the growing demand for chicken meat. This meta-analysis comprehensively assesses IBDV prevalence and offers valuable insights to support effective disease control strategies and informed management of chicken operations.

In this meta-analysis, all *p-values* were below 0.05, indicating significant publication bias (Table 2). The right side of the funnel plot showed an asymmetrical distribution, with more data points on the right side (indicating higher prevalence) than on the left side (lower prevalence). This imbalance may be attributed to the smaller standard deviations and standard errors in large-sample studies, which cluster above the vertical axis at the top of the funnel plot, particularly in the upper right corner when the effect size is positive. Large-sample studies are often perceived as more methodologically sound and yield more reliable results. Moreover, farms in high prevalence regions may be more motivated to participate in research and contribute larger sample sizes, leading to data clustering in those areas (15). This suggests that studies reporting higher IBDV prevalence, especially those with large samples, are more likely to be published. Alternatively, high-prevalence regions may necessitate larger samples to confirm findings, further skewing the dataset (16). Furthermore, geographic or temporal factors may influence publication trends, as high prevalence areas often draw more research attention, contributing to the overrepresentation of such regions in the data.

This review examined 32 studies published between 1983 and 2024, encompassing data from 210,983 chickens in China, and found an overall IBDV prevalence of 37%, a rate somewhat lower than those reported in several other countries. While the highest prevalence in Northwest China is reported to be 88%, this study's investigation was limited to Ningxia Province. Consequently, the limited study in this region and incomplete analysis prevent it from fully representing the complex situations across the entire northwest region and other provinces, which introduce certain limitations (17). Infectious bursal disease is specially prevalent in countries with highly developed chicken industries, including the United Kingdom, France, Denmark, the Netherlands, Germany, the United States, India and Canada (18). For example, a 2016 Canadian study showed that the seroprevalence of IBDV in 1,059 commercial chickens was 84% (19), while in India, the seroprevalence in 400 chickens was 73.75% (20). This could be attributed to the more complex epidemiological landscape of IBDV serotypes and variants in developed countries and the higher exposure risk of intensive, large-scale, chicken farming, facilitating rapid viral transmission within chicken populations (21, 22).

In chicks under 1 week of age, maternal antibodies neutralize viruses in the IBD, rendering them undetectable before 2-week-old, causing serological tests to overestimate the positive rate, and making it difficult to exclude maternal antibody interference via standard antibody detection methods (23). Chicks aged 3–6 weeks are susceptible to IBD, often presenting with severe acute symptoms and high mortality, whereas those aged 0–3 weeks mostly develop mild acute symptoms or subclinical disease after infection (24). Zhang et al. (25) also observed that adult chickens may become more susceptible when their immune function is compromised by other factors, a finding that aligns with the results of our study. Furthermore, Hurisa et al. (26) identified the presence of multiple IBDV serotypes and variants, combined with high-density chicken populations and poor ventilation, as major contributors to infection risk.

The increased susceptibility of laying chickens may be due to the anatomical structure of the bursa tissue in certain their longer feeding cycles, which make them more vulnerable to viral invasion (27). For example, Hair-Bejo et al. reported that emerging IBDV strains caused mortality rates ranging from 10% to 75% in layer chickens and 10% to 40% in broiler chickens. In the early 2000s, layer chickens were more vulnerable to IBDV than broilers, a finding that aligns with the results of our study (28). Interestingly, while layer chickens showed higher positivity rates than broiler chickens, they were more likely to be infected with variant strains, with some positivity rates reaching up to 39% (29). Because the bursa of Fabricius in layer chickens features more complex mucosal folds, with the number of lymphoid follicles being 1.2–1.5 times that in broilers and a higher concentration of B cells within these follicles, making it a more ideal replication site for IBDV (30). Tippenhauer et al. (31) explored the immunopathogenesis of IBDV in both layer chickens and broiler chickens. They found that layer chickens of various genetic lines consistently showed significantly higher (*p* < 0.05) IBDV antigen loads in the bursa of Fabricius compared to broiler chickens.

Seasonal patterns play a key role in the infectious bursal disease virus epidemiology, with infections occurring year round but more commonly concentrated between March and November, particularly during significant weather fluctuations (32). In this study, IBDV prevalence was significantly higher in spring and autumn, at 39% (95%CI: 2–80) and 55% (95%CI: 45–64), respectively, while summer and winter showed slightly lower rates of 35% (95%CI: 10–59) and 38% (95%CI: 8–67). These findings support that IBDV is transmitted throughout the year, with seasonal peaks. Study by, Adino et al. (33), reported the samples collected in spring and autumn showed a higher incidence of IBD detection than those gathered in summer. This suggests that seasonal fluctuations in temperature and humidity may influence the environmental stability of IBDV and contribute to respiratory and gastrointestinal vulnerability in newly hatched chicks. These seasonal effects may also reflect climatic differences between countries. Abey et al. (34) similarly highlighted the significant role of seasonal variation in influencing IBDV infection dynamics. These findings align with previous studies in China that have documented the potential impact of seasonal factors on IBDV prevalence (35).

The sensitivity of IBDV detection methods, including AGP, ELISA, and RT-PCR, varied indicating a potential association between assay type, sample characteristics, and observed prevalence. In this study, the positive detection rates of AGP, ELISA, and RT-PCR were 34%, 41%, and 32%. The ELISA method is widely used to detect antibodies after vaccination due to its high sensitivity, and is capable of effectively monitoring immune responses, even detecting antibodies at low concentrations (36). In addition, the high specificity of ELISA reagents ensures the accuracy of test results and avoids cross-reactions with other viral antibodies (37). The combination of ELISA and PCR methods provides a comprehensive, efficient, and reliable solution for IBDV detection. ELISA is used for rapid screening, while PCR is used for accurate confirmation and typing. The complementary nature of these two methods makes them extremely valuable in virus detection and epidemiological research (38). For instance, Saravanan et al. (39) reported that most studies used serological methods based on enzyme-linked ELISA, which are relatively simple to operate and suitable for large-scale sample testing, consistent with the methods used in this analysis. The application of ELISA kits enables reliable monitoring of IBDV prevalence, facilitates accurate monitoring of immune responses, and supports the development of effective immunization and control strategies to prevent IBDV (40).

Farming practices also influence the overall incidence of IBD in chicken populations. In this study, the IBD positivity rate was 45% (95% CI: 24–66) in chickens raised on intensive chicken farm and 42% (95% CI: 25–58) in free-range chickens. Rong et al. (41) similarly found that intensive chicken farm were more likely to report IBDV infections than free-range systems, likely due to the higher density of chickens that facilitates rapid viral transmission. However, as Shekhar et al. (42) noted, free-range chickens are also vulnerable to IBDV, and underdiagnosis remains a concern due to limited access to diagnostic tools and insufficient disease awareness among farmers. Understanding the differences in IBD prevalence across farming systems is crucial for designing targeted prevention and control strategies that effectively reduce the impact of the disease on the chicken populations (15).

The application of traditional IBDV vaccines in Chinese chicken populations faces severe challenges, mainly due to the emergence of new variants and genetic diversity. Although traditional vaccines have achieved significant results in controlling classic strains and attenuated strains, their protective effect against new variants is limited (16). Therefore, developing vaccines targeting new variants has become a matter of urgency. Wang et al. (16) reported that combining traditional vaccines with booster immunization strategies can improve protection against new variants to a certain extent. However, further research is needed on the relationship between viral genotypes and antigenicity in order to design more effective vaccines. In addition, the genetic recombination and reassortment phenomena of IBDV indicate that the virus evolves rapidly and may give rise to more new variants in the future (43). Therefore, strengthening virus surveillance and genotyping is crucial for timely adjustment of vaccine strategies. At the same time, exploring novel vaccine technologies based on viral proteins (such as VP2 and VP5) may provide new solutions for IBDV control (44). The vaccination status and genotype data of traditional IBDV in Chinese chicken populations reflect the complex relationship between viral evolution and vaccine protection. In the future, it will be necessary to combine genotype analysis and new vaccine technologies to address the ever-changing viral threats.

### 4.1 Limitations

This meta-analysis provides a comprehensive overview of IBDV infections in chicken populations across China, highlighting regional variations influenced by geographical and climatic factors. It should be noted, however, that despite efforts to ensure the accuracy and reliability of the results, this study is still subject to certain limitations. First, although data were obtained from six major databases, not all sources yielded relevant studies, potentially contributing to the limited availability of eligible literature. Second, the relatively small sample sizes in the included studies may have introduced variability in the overall prevalence estimates and subgroup analyses. Third, key risk factors such as viral serotypes and strains, maternal antibodies, and co-infections could not be thoroughly assessed due to insufficient data. Future studies should address these gaps to enhance understanding of IBDV epidemiology and support the development of targeted prevention and control strategies across mainland China.

## 5 Conclusion

In conclusion, this meta-analysis revealed an overall IBDV prevalence of 37% among chicken populations in China, with the highest rate of 88% reported in northwestern regions. Significant regional and provincial variations were observed, and several factors, including growth stage, type, farming scale, and season, were identified as important contributors to IBDV infection risk. Furthermore, differences in detection method may influence the reported prevalence. These findings provide valuable insights for veterinary practice, and inform targeted disease control strategies and policy development to reduce the burden of IBDV in China's populations industry.

## Data Availability

The original contributions presented in the study are included in the article/supplementary material. For access to the complete original data to replicate the study results, please contact the corresponding author.
